# Knockdown of ELF4 aggravates renal injury in ischemia/reperfusion mice through promotion of pyroptosis, inflammation, oxidative stress, and endoplasmic reticulum stress

**DOI:** 10.1186/s12860-023-00485-2

**Published:** 2023-07-20

**Authors:** Li Li, Shunying Wang, Wenming Wang

**Affiliations:** 1 Department of Nephrology, Jinan City People’s Hospital, No. 001, Changshao North Road, Laiwu District, Jinan, Shandong 271199 People’s Republic of China; 2 Department of Cadre Health Section, Jinan City People’s Hospital, Jinan, Shandong 271199 People’s Republic of China

**Keywords:** Acute kidney injury, ELF4, Inflammation, Pyroptosis, Oxidative stress

## Abstract

**Background:**

Renal ischemia/reperfusion (I/R) injury is a major cause of acute kidney injury (AKI). Dysfunction of E74-like ETS transcription factor 4 (ELF4) leads to inflammation. This research intended to look into the function and mechanisms of ELF4 in I/R and oxygen–glucose deprivation/reperfusion (OGD/R) model.

**Results:**

In I/R and OGD/R model, ELF4 expression was downregulated. ELF4 knockout aggravated I/R-induced kidney injury, oxidative stress (OS), endoplasmic reticulum stress (ERS), apoptosis, inflammation, and pyroptosis in mice. In HK-2 cells treated with OGD/R, suppression of ELF4 expression inhibited cell proliferation and promoted cell apoptosis, OS, ERS, inflammation, and pyroptosis. Moreover, ELF4 overexpression led to the opposite results.

**Conclusion:**

ELF4 deficiency aggravated I/R induced AKI, which was involved in apoptosis, OS, ERS, inflammation, and pyroptosis. Targeting ELF4 may be a promising new therapeutic strategy for preventing inflammation after IR-AKI.

**Supplementary Information:**

The online version contains supplementary material available at 10.1186/s12860-023-00485-2.

## Background

As a major kidney disease, acute kidney injury (AKI) is a clinical syndrome characterized by sudden loss of renal function with sublethal renal tubular injury [1]. Clinically, renal ischemia/reperfusion (I/R) injury is a major cause of AKI, usually occurring in hypovolemic shock, surgery, sepsis, trauma, and kidney transplantation [2–4]. Although AKI is paid close attention for the higher morbidity and mortality rates, AKI is still a difficult problem in diagnosis and treatment in clinic [5]. AKI pathogenesis is complicated, which is related to abnormal apoptosis, oxidative stress (OS), endoplasmic reticulum stress (ERS), inflammatory responses, and pyroptosis [6–10]. However, the exact mechanisms of AKI remains poorly understood. Therefore, exploring the potential mechanism of AKI is of great significance for treatment of AKI.

The E-Twenty-Six (ETS) transcription factor family is composed of 29 members in human and 28 members in mouse, which participates in various signaling pathways [11–13]. E74 like ETS transcription factor 4 (ELF4), originally called "myeloid Elf1-like factor", has a hand in tumorigenesis, regulating immune responses, DNA damage response, and cell cycle regulation [14–17]. Lee et al. have indicated that ELF4 knockout (KO) in mice contributes to the increased disease severity after experimental autoimmune encephalomyelitis induction [18]. Du et al. have shown that ELF4 KO mice are sensitive to dextran sulfate sodium-induced salt colitis [19]. Moreover, ELF4 is reported to restrain inflammation and protecte against mucosal disease [20]. However, the influence of ELF4 is unclear in AKI. Therefore, studying the influence of deficiency of ELF4 on renal injury is necessary.

At the present study, in vivo model induced by I/R and in vitro model induced by oxygen–glucose deprivation/reperfusion (OGD/R) were used for determining the influences and mechanisms of ELF4 in AKI. Mice lacking ELF4 showed worsened kidney structure and function after I/R. Subsequent studies revealed a direct effect of ELF4 in protecting HK-2 cells from apoptosis, OS, ERS, inflammation, and pyroptosis. A new target for AKI treatment may obtain from our findings.

## Results

### In kidney tissues of I/R mice and cells of OGD/R, ELF4 expression is decreased

**Fig. 1:**
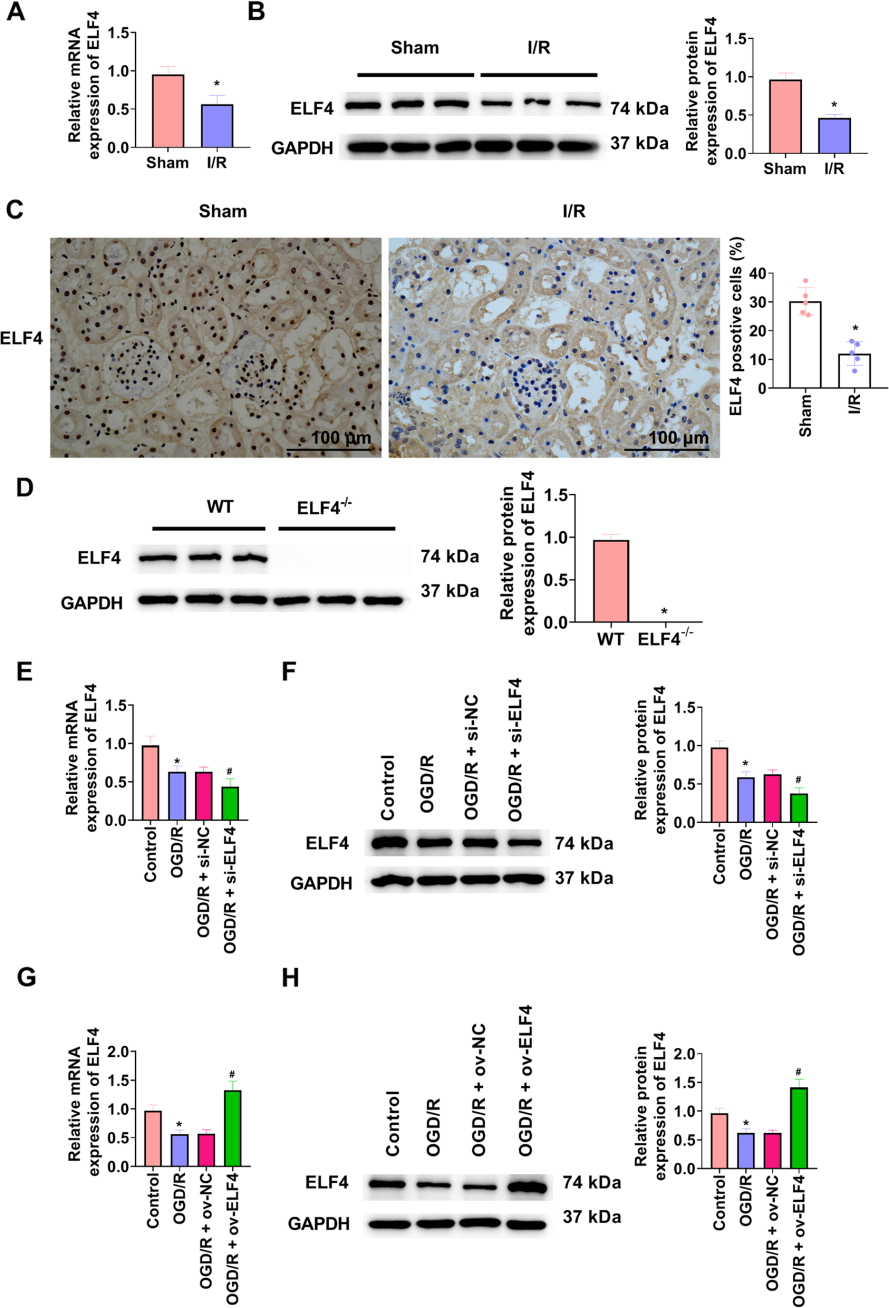
In kidney tissues of I/R mice and cells of OGD/R, ELF4 expression was decreased. **A** ELF4 mRNA expression was detected by way of qRT-PCR in kidney tissues of sham and I/R mice. ELF4 protein expression in kidney tissues of sham and I/R mice was surveyed using western blot (**B**) and immunohistochemistry (**C**). **D** In kidney tissues of WT and ELF4^−/−^ mice, ELF4 protein change was ascertained using western blot. **E** ELF4 mRNA expression was surveyed by way of qRT-PCR in HK-2 cells. **F** In HK-2 cells, western blot was applied to survey ELF4 protein expression. **G** ELF4 mRNA expression was surveyed by way of qRT-PCR in HK-2 cells. **H** In HK-2 cells, ELF4 protein expression was checked by way of western blot. Mouse *n* = 5. Cell *n* = 3. * *p* < 0.05 vs sham, WT, or control group; # *p* < 0.05 vs OGD/R group

To explore whether ELF4 involved in kidney injury, we detected the levels of ELF4. Figure 1A showed that ELF4 mRNA expression was markedly decreased in kidney tissues of I/R mice. Furthermore, ELF4 protein expression was significantly reduced, which was indicated by western blot and immunohistochemical analysis (Fig. 1B and C). ELF4^−/−^ mice were applied to investigate the function of ELF4 in I/R mice (Fig. 1D). In vitro, HK-2 cells were simulated with OGD/R to mimic I/R. To look into the influence of ELF4 in cell model of renal injury, ELF4 siRNA and overexpression plasmid were transfected into HK-2 cells. OGD/R significantly reduced ELF4 expression, transfection of ELF4 siRNA further markedly reduced ELF4 expression, and transfection of ELF4 overexpression plasmid significantly induced ELF4 expression (Fig. 1E-H).

### In mice, ELF4 knockout aggravates I/R-induced kidney injury

**Fig. 2:**
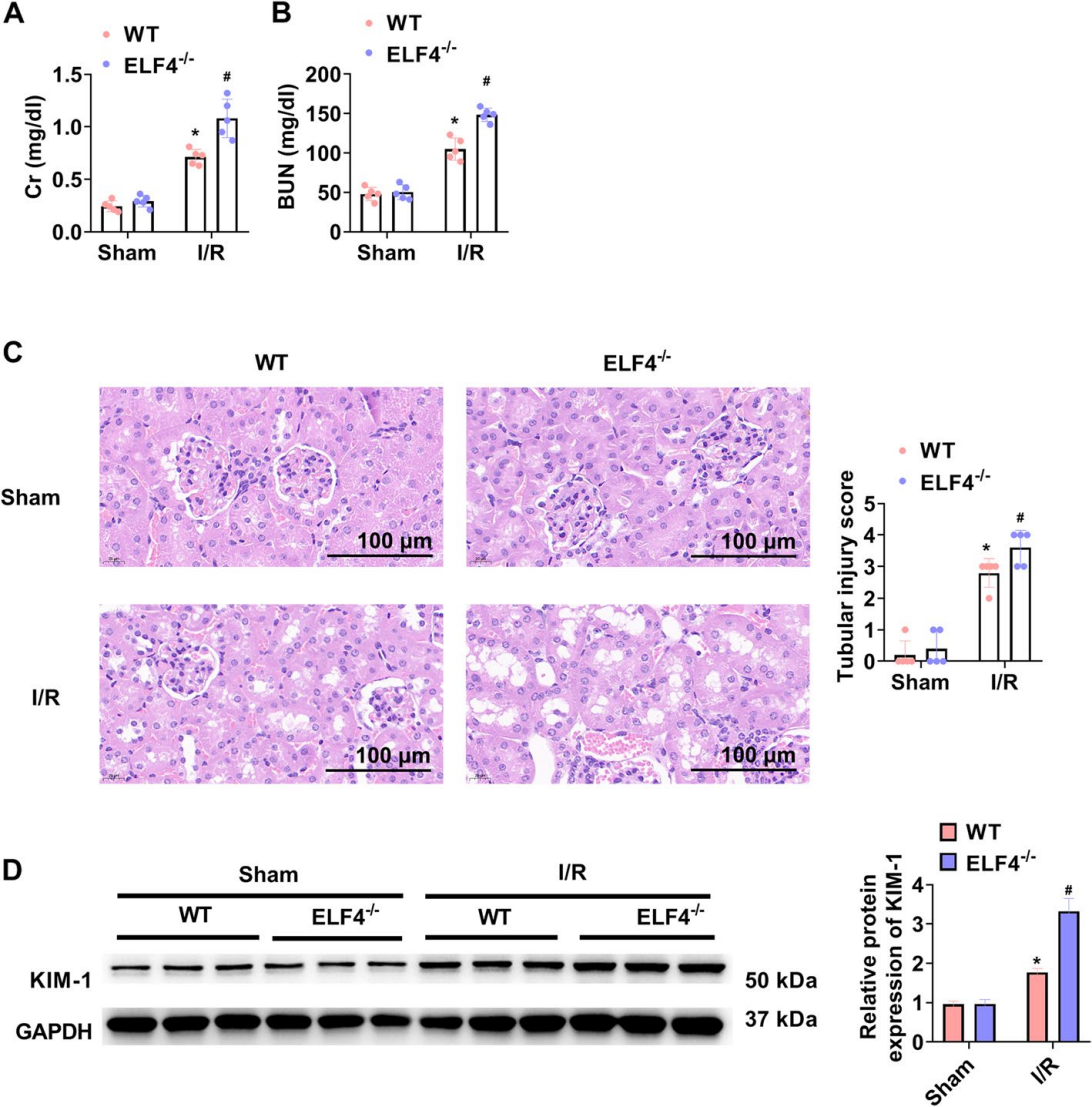
In I/R mice, ELF4 deficiency aggravated kidney injury. Serum Cr (**A**) and BUN levels (**B**) in WT and ELF4^−/−^ mice. **C** In WT and ELF4^−/−^ mice, the renal histological injury was estimated by way of H&E staining. **D** KIM-1 protein change was ascertained in kidney tissues of WT and ELF4^−/−^ mice using western blot. Mouse *n* = 5. * *p* < 0.05 WT sham group, # *p* < 0.05 ELF4^−/−^ sham or WT I/R group

Figure 2A and B suggested that serum Cr and BUN levels were low in both ELF4^−/−^ and WT mice of sham group, while serum Cr and BUN levels were markedly increased in both ELF4^−/−^ and WT mice of I/R group. Moreover, in I/R group, ELF4^−/−^ further obviously increased serum Cr and BUN levels. H&E staining showed that both ELF4^−/−^ and WT mice of sham group had normal kidney structure, after renal I/R, both ELF4^−/−^ and WT mice showed swollen, extensive expansion and deformation of renal tubules. In I/R group, ELF4^−/−^ further aggravated tubular injury (Fig. 2C). It has shown in rats of I/R injury model that KIM-1 expression in the proximal tubule is induced by ischemia [21]. I/R significantly enhanced KIM-1 expression, and ELF4^−/−^ further enhanced KIM-1 expression in I/R group (Fig. 2D).

### ELF4 deficiency aggravates apoptosis

**Fig. 3:**
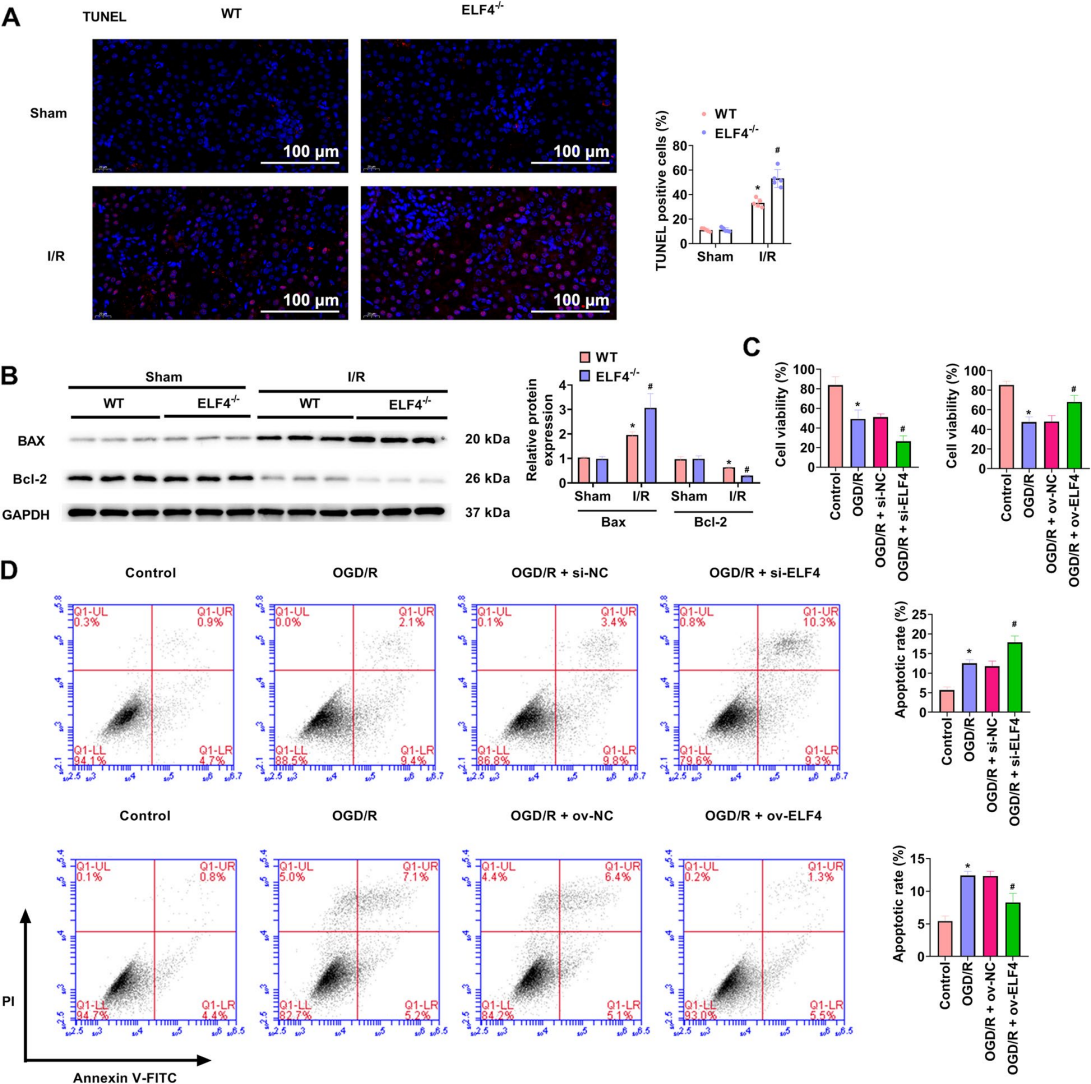
ELF4 deficiency aggravated apoptosis. **A** In WT and ELF4-/- mice, TUNEL staining was applied to survey renal cell apoptosis. **B** Bax and Bcl-2 protein levels were detected by way of western blot in kidney tissues of WT and ELF4-/- mice. **C** HK-2 cell viability was ascertained by way of CCK-8 assay. **D** Flow cytometry was carried out to assess cell apoptosis in HK-2 cells. Mouse *n* = 5. Cell *n* = 3. * *p* < 0.05 WT sham or control group, # *p* < 0.05 ELF4^−/−^ sham, WT I/R, or OGD/R group

TUNEL staining was used to evaluate apoptosis in I/R mouse model. TUNEL staining showed that cell apoptosis was markedly increased by I/R, and ELF4^−/−^ further increased cell apoptosis (Fig. 3A). Bax, a proapoptotic member of the Bcl-2 family. Bcl-2 exerts a death-sparing activity against apoptosis induced by I/R [22]. I/R obviously promoted Bax expression and significantly inhibited Bcl-2 expression in renal tissues, and ELF4^−/−^ exacerbated these changes (Fig. 3B). As indicated in Fig. 3C and D, OGD/R significantly inhibited cell viability and markedly promoted cell apoptosis, suppression of ELF4 expression further aggravated these effects induced by OGD/R. Overexpression of ELF4 markedly eliminated the influence of OGD/R on cell viability, apoptosis.

### ELF4 deficiency aggravates OS and ERS

**Fig. 4:**
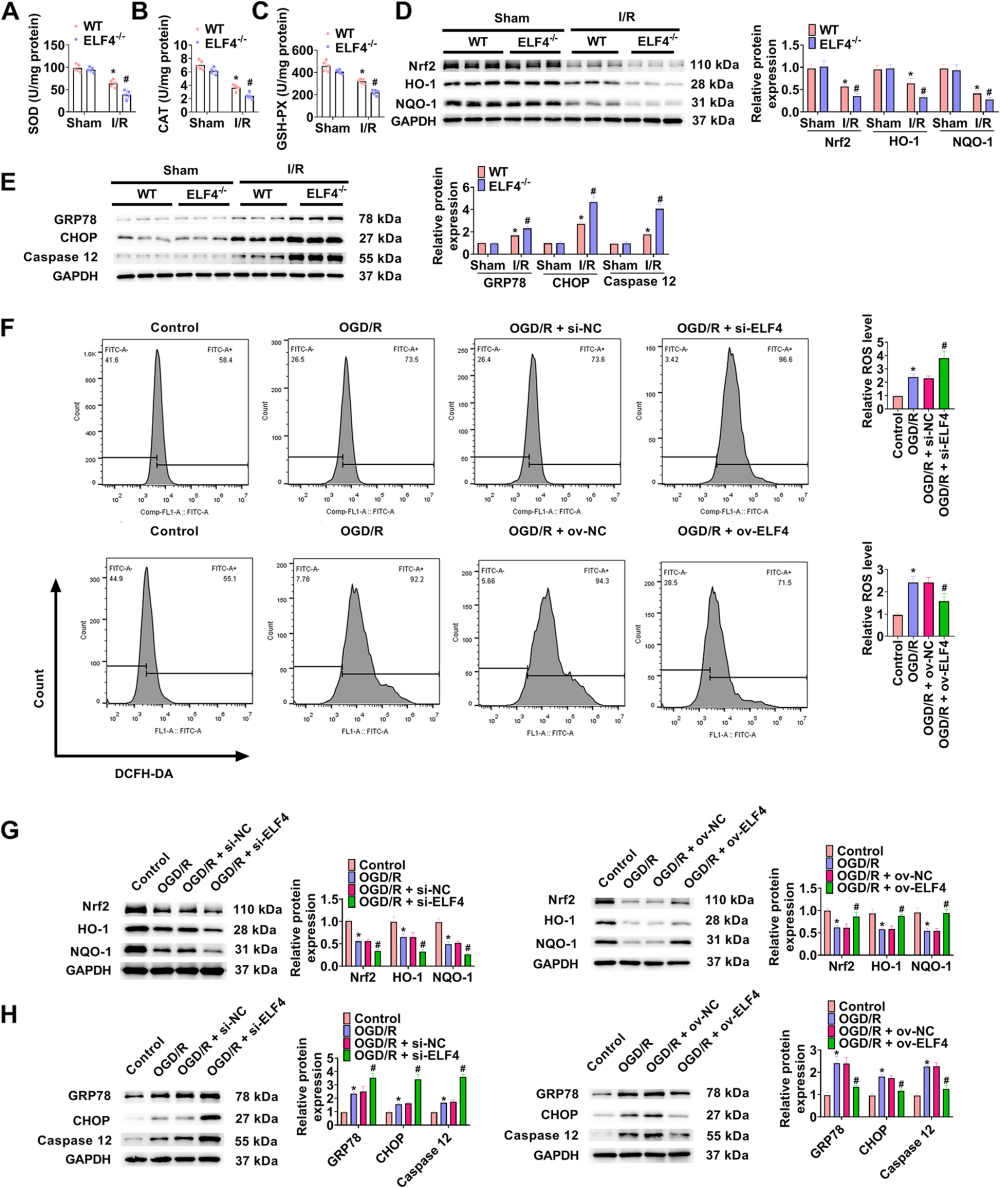
ELF4 deficiency aggravated OS and ERS. Serum SOD (**A**), CAT (**B**), and GSH-PX (**C**) levels in WT and ELF4^−/−^ mice. **D** In kidney tissues of WT and ELF4^−/−^ mice, Nrf2, HO-1, and NQO-1 protein levels were surveyed by way of western blot. **E** GRP78, CHOP, and caspase-12 protein levels were detected by way of western blot in kidney tissues of WT and ELF4^−/−^ mice. **F** Flow cytometry was carried out to assess ROS levels in HK-2 cells. The levels of OS related proteins (Nrf2, HO-1, and NQO-1; **G**), ER stress related proteins (GRP78, CHOP, and caspase-12; **H**) were surveyed using western blot in HK-2 cells. Mouse *n* = 5. Cell *n* = 3. * *p* < 0.05 WT sham or control group, # *p* < 0.05 ELF4^−/−^ sham, WT I/R, or OGD/R group

In I/R induced kidney injury, to understand the involvement of ELF4 and whether this influences were related to OS and ERS, we assessed the change of OS index (SOD, CAT, and GSH-PX), OS related proteins (Nrf2, HO-1, and NQO-1), and ERS related proteins (GRP78, CHOP, and caspase-12). Figure 4A-C presented that I/R markedly reduced serum SOD, CAT, and GSH-PX levels. In I/R group, ELF4^−/−^ further reduced serum SOD, CAT, and GSH-PX levels. A obvious reduction of protein levels of Nrf2, HO-1, and NQO-1 was showed in renal tissues of I/R group, after renal I/R, ELF4^−/−^ further aggravated these protein change (Fig. 4D). In addition, I/R caused an obvious increase of GRP78, CHOP, and caspase-12 protein in renal tissue, and ELF4^−/−^ further increased these changes of proteins (Fig. 4E). Figure 4F showed that ROS level was obviously increased by OGD/R, suppression of ELF4 further enhanced ROS level, and overexpression of ELF4 significantly decreased ROS level. Consistently, OGD/R markedly inhibited Nrf2, HO-1, and NQO-1 protein levels, and suppression of ELF4 further aggravated the inhibition role (Fig. 4G). In addition, OGD/R obviously increased the levels of GRP78, CHOP, caspase-12, and suppression of ELF4 further aggravated the inhibition role (Fig. 4H). Moreover, western blot analysis (Fig. 4G-H) showed that overexpression of ELF4 significantly increased Nrf2, HO-1, and NQO-1 protein levels, and markedly decreased GRP78, CHOP, caspase-12.

### ELF4 deficiency aggravates inflammation and pyroptosis

**Fig. 5:**
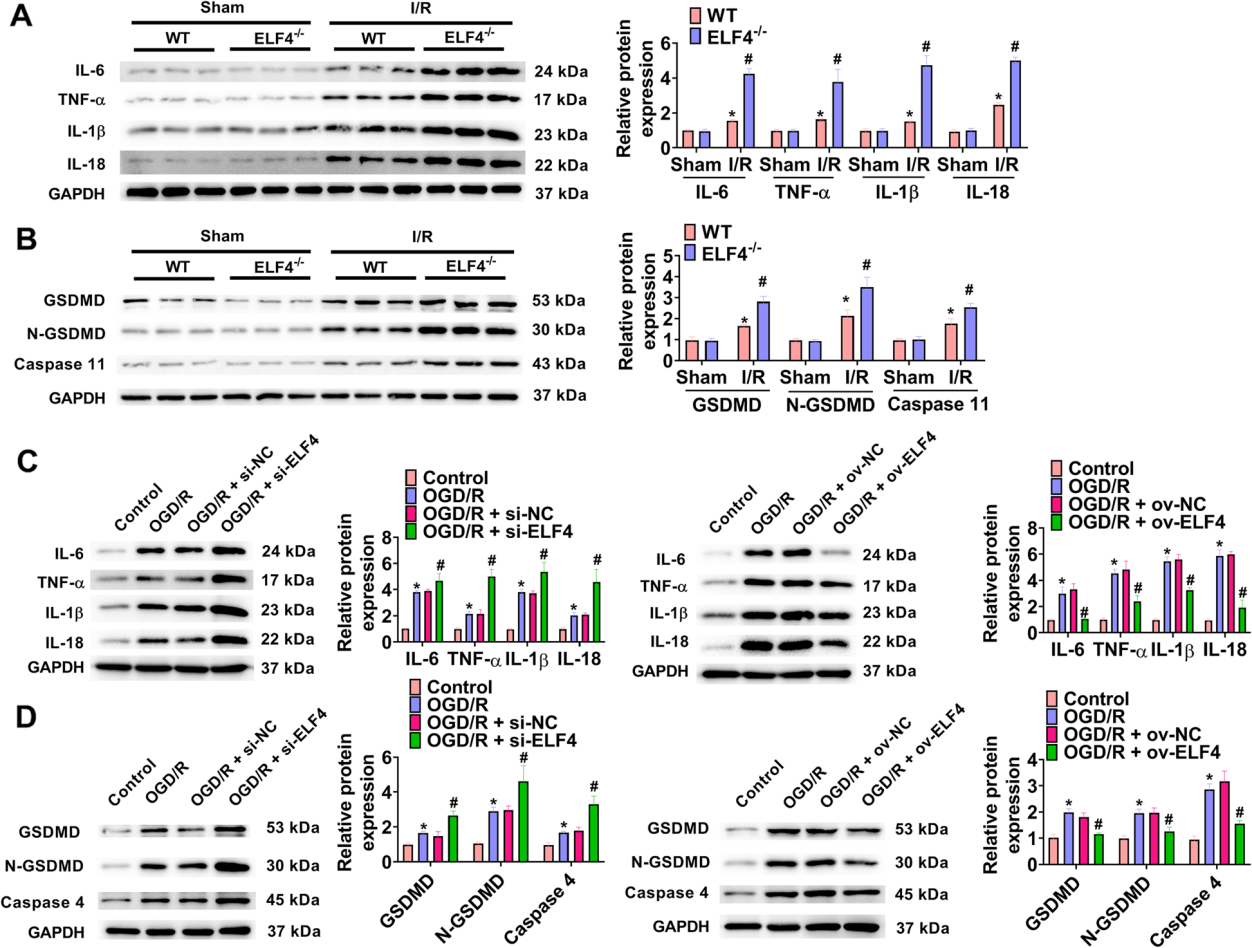
ELF4 deficiency aggravated inflammation and pyroptosis. **A** In kidney tissues of WT and ELF4^−/−^ mice, IL-6, TNF-α, IL-18, and IL-1β protein levels were detected by way of western blot. **B** GSDMD, N-GSDMD, and caspase-11 protein levels were surveyed in kidney tissues of WT and ELF4^−/−^ mice using western blot. The levels of inflammatory cytokines (IL-6, TNF-α, IL-18, and IL-1β; **C**), and pyroptosis-related proteins (GSDMD, N-GSDMD, and caspase-11; **D**) were detected using western blot in HK-2 cells. Mouse *n* = 5. Cell *n* = 3. * *p* < 0.05 WT sham or control group, # *p* < 0.05 ELF4^−/−^ sham, WT I/R, or OGD/R group

Subsequently, the influence of ELF4 on inflammatory cytokines was investigated. Figure 5A showed that I/R markedly increased the levels of IL-6, TNF-α, IL-18, and IL-1β, and these inflammatory cytokines were further increased by ELF4 deficiency in I/R groups. Furthermore, in renal tissues, I/R significantly enhanced pyroptosis related proteins (GSDMD, N-GSDMD, and caspase-11) protein levels, and these proteins in renal tissues of I/R group was further increased following ELF4 deficiency (Fig. 5B). Moreover, in OGD/R-treated cells, western blot analysis showed that ELF4 knockdown or overexpression significantly increased or decreased IL-6, TNF-α, IL-18, IL-1β, GSDMD, N-GSDMD, and caspase-4 protein levels (Fig. 5C-D).

## Discussion

In clinic, renal ischemia that occurs in kidney surgery, transplantation, hemorrhagic, cardiogenic, septic shock, and other clinical environments is the most common cause of AKI [4, 23]. Renal I/R is generally acknowledge as an important cause of AKI mortality, especially among patients in ICU [24–26]. At present, there is still no available treatment to prevent ischemic injury, and there is no therapy or drug that can completely reduce I/R induced mortality [27–29]. Thus, searching for effective therapeutic approaches for renal I/R injury is an urgent need. In this study, I/R and OGD/R induced AKI model were established. We found that ELF4 expression was markedly downregulated in I/R and OGD/R induced model, which indicated that ELF4 might take part in the pathological process of AKI.

In I/R and OGD/R model, we restrained ELF4 expression to explore the effects of ELF4 in AKI. The most commonly used markers for renal function assessment including Cr and BUN [30]. The levels of serum Cr and BUN were found to be increased in I/R mice [31–33]. As a transmembrane glycoprotein, KIM-1 can be split into soluble fragments and eventually excreted into urine [34, 35]. Therefore, KIM-1 is often used as a biomarker of renal injury [36]. Our data demonstrated that inhibition of ELF4 exacerbated kidney damage, as evidenced by the increase of serum Cr, serum BUN, KIM-1 protein expression in renal tissue. In AKI, the main pathways of cell death are apoptosis and necrosis [37, 38]. In this study, in I/R mice and HK-2 cells treated with OGD/R, inhibition of ELF4 expression markedly increased cell apoptosis. Taken together, inhibition of ELF4 expression aggravated renal injury.

The pathogenesis of AKI associated with I/R is involved in tubular damage and inflammatory response [23]. More and more evidence showed that I/R promoted the release of inflammatory factors in renal tissue, which leads to severe renal cell apoptosis, thereby causing AKI [39–41]. For ameliorating AKI and facilitating recovery, inhibition of inflammatory response is a promising therapeutic approach [42]. As an important inflammatory factor, IL-1β takes part in the process of hosts against pathogens [43]. IL-18 is mainly produced by activated mononuclear macrophages, which participates in ischemic AKI [44]. Moreover, other inflammatory cytokines, for instance, IL-6 and TNF-α can be elevated by IL-1β and IL-18 [45]. At the present work, I/R and OGD/R markedly increased the levels of IL-6, TNF-α, IL-1β, and IL-18. Meanwhile, in I/R and OGD/R model, inhibition of ELF4 further increased IL-6, TNF-α, IL-18, and IL-1β levels, but overexpression of ELF4 led to the opposite results. Doitsh et al. have reported that pyroptosis generated pro-inflammatory mediators, thereby triggering inflammatory reactions [46]. After that, we detected the changes of pyroptosis-related proteins, and found that inhibition of ELF4 further increased GSDMD, N-GSDMD, and caspase-11 levels in I/R and OGD/R model. Furthermore, in OGD/R model, GSDMD, N-GSDMD, and caspase-11 levels were markedly reduced by overexpression of ELF4. In short, in pathogenesis of AKI, inhibition of ELF4 promoted inflammatory response and pyroptosis.

The accumulation of ROS during I/R process is vital in the course of AKI [47]. After reperfusion, the first injury event is that mitochondria produce ROS, secondary tissue damage and subsequent inflammation caused by non-mitochondrial ROS [48]. Common biochemical markers of oxidative damage include SOD, CAT, and GSH-PX [49]. In OGD/R model, inhibition of ELF4 significantly increased ROS levels, but overexpression of ELF4 dramatically decreased ROS levels. In I/R model, inhibition of ELF4 markedly reduced SOD, CAT, and GSH-PX levels. Moreover, in I/R and OGD/R model, inhibition of ELF4 significantly inhibited the expression of OS related proteins (Nrf2, HO-1, and NQO-1). Apart from regulating OS, it was indicated that Nrf2/HO-1 pathway had a hand in regulating apoptosis, ERS, and inflammation [50–52]. To further investigate the mechanism of ELF4 in AKI, ERS related proteins (GRP78, CHOP, and Caspase-12) were detected. Our findings indicated that ELF4 inhibition significantly promoted ERS in I/R and OGD/R model, as evidenced by increasing GRP78, CHOP, and caspase-12 levels. Based on these findings, inhibition of ELF4 aggravated renal injury, which was associated with the OS and ERS.

In summary, inhibition of ELF4 aggravated renal injury in I/R treated mice by promoting OS, ERS, inflammation, and pyroptosis. In future studies, ELF4 supported the transcription of OS, ERS, inflammation, and pyroptosis-related gene will be studied in I/R-induced AKI. Our findings may provide a novel mechanistic insight into I/R-induced AKI.

## Methods

### Mouse model of renal I/R injury

The male ELF4 knockout (ELF4^−/−^) and wild-type (WT) C57BL/6 mice were provided by Beijing Vital River Laboratory Animal Technology Co., Ltd. (China). In a 12 h light/12 h dark cycle, eating and drinking could be freely obtained by mice. The animal care and use committee of our hospital authorized all experiments.

ELF4^−/−^ mice and WT mice were randomly assigned to sham and I/R group, respectively. AKI model was induced by I/R surgery [53]. In brief, after anesthesia with 1% sodium pentobarbital solution, mice were underwent a midline laparotomy. Next, a microaneurysm clamp (Fine Science Tools, USA) was used to tighten both renal vessels for 30 min to induce ischemia. After removing the clamp for 6 h, the serum was collected from mice before sacrificing. In sham group, mice were exposed to the same procedure, except using the microaneurysm clamp.

### Cell culture and treatments

Human proximal tubular epithelial cells (HK-2) were provided by Procell Life Science&Technology Co.,Ltd (CL-0109, Wuhan, China). Under condition of 37 °C and 5% CO_2_, cells were maintained in DMEM (11885084, Invitrogen, USA) with 1% penicillin/streptomycin and 10% foetal bovine serum.

Small interference RNA targeting ELF4 (si-ELF4), overexpression plasmid of ELF4 (ov-ELF4), and negative control (si-NC and ov-NC) were transfected into HK-2 cells by way of Lipofectamine 2000 reagents (11668030, Invitrogen). After transfection for 48 h, cells in OGD/R + si-NC, OGD/R + si-ELF4, OGD/R + ov-NC, and OGD/R + ov-ELF4 group were treated with OGD/R. To establish OGD/R model, HK-2 cells were exposed to glucose-free medium supplemented with 1% O_2_, 5% CO_2_, and 94% N_2_ for 4 h at 37 °C [10]. Next, the complete medium with 21% O_2_ was used for maintaining cells for 6 h. In control group, cells were maintained in complete medium with 21% O_2_.

### Quantitative real-time polymerase chain reaction (qRT-PCR)

In kidney tissues and HK-2 cells, total RNA was extracted by means of TRIzol reagent (15596026, Invitrogen, USA). The PrimeScript™ RT Reagent Kit (RR037Q, Takara, Japan) was made use of synthesizing complementary DNA. The SYBR Premix Ex Tag Kit (RR820A, Takara) was made use of performing qRT-PCR. The sequences of primers were listed in Supplementary table 1.

### Western blot

In kidney tissues and HK-2 cells, RIPA lysis buffer (20–188, Sigma-Aldrich) was used for extracting proteins. An equal quantity of protein was electrophoresed using SDS-PAGE and then transferred onto PVDF membranes. At 4 °C, primary antibodies against ELF4, Nrf2, NQO-1, GRP78, KIM-1, and IL-6 (1:1000, ab96075, ab137550, ab34173, ab21685, ab302932, and ab259341, Abcam, USA); IL-1β (1:1000, sc-12742, Santa Cruz Biotechnology); IL-18 and N-GSDMD (1:1000, A1115 and A22523, ABclonal, USA); Bax, Bcl-2, TNF-α, caspase-4, caspase-11, Caspase-12, CHOP, HO-1, and GSDMD (1:1000, 2772, 3498, 3707, 4450, 14340, 35965, 2895, 43966, and 39754, Cell signaling Technology, USA), and GAPDH (1:5000, 5174, Cell signaling Technology, USA) were deal with membranes overnight. After that, secondary antibodies (1:50000, 7074, Cell signaling Technology) were handled with membranes for 1 h. At last, protein bands were visualized by way of an enhanced chemiluminescence detection kit (Beyotime Biotechnology, China). Image J software (USA) was used to quantify protein expression.

### Immunohistochemistry analysis

The kidney tissues of mice were fixed in 10% formalin (HT501128, Sigma-Aldrich). After that, paraffin was taken to embed kidney samples. After dewaxing and rehydrating, Sects. (4 μm-thick) were incubated with 3% hydrogen peroxide (H1009, Sigma-Aldrich) for 10 min. Next, sections were blocked with 5% bovine serum albumin (A1933, Sigma-Aldrich) for 1 h and incubated with antibody against ELF4 (1:200, bs-14563R, Bioss) 4˚C. Nexy day, sections were incubated with secondary antibody (8114, Cell signaling Technology). At last, sections were developed color using 3,3’-diaminobenzidine (P0202, Beyotime Biotechnology). Under a microscopy (BX53, Olympus, Japan), ELF4 positive cells were quantified.

### Haematoxylin and Eosin (H&E) staining

After dewaxing and rehydrating, the renal Sects. (4 μm-thick) were stained with haematoxylin (C0107-100 ml, Beyotime Biotechnology) for 5 min and stained with eosin (C0109, Beyotime Biotechnology) for 1 min. Under an optical microscope (Olympus), renal tissue damage was assessed in a blinded manner.

### Terminal deoxynucleotidyl transferase dUTP nick end labeling (TUNEL) staining

In kidney tissues, cell apoptosis was ascertained by way of the TUNEL apoptosis detection kit (C1089, Beyotime Biotechnology). Briefly, after dewaxing and rehydrating, the renal sections were treated with proteinase K without DNase (20 µg/ml) for 20 min. Next, sections were stained with the TUNEL reaction mixture, followed by staining with DAPI. Eventually, under an optical microscope, apoptotic cells were observed and counted.

### Biochemical assays

For the detection of serum creatinine (Cr, C011-2–1), blood urea nitrogen (BUN, C013-2–1), superoxide dismutase (SOD, A001-1–2), catalase (CAT, A007-1–1), and glutathione peroxidase (GSH-PX, A005-1–2), the serum was collected from mice blood through centrifuging at 3,000 rpm for 10 min. Nanjing jiancheng bioengineering institute (China) provided corresponding kits.

### Detection of cell viability

After indicated treatment, cell counting kit 8 (CCK-8) reagents (C0037, Beyotime Biotechnology) were applied to handle HK-2 cells. After handling for 2 h, at 450 nm, a BioTek ELX-800 microplate reader (USA) was utilized to gauge the optical density (OD) value.

### Detection of cell apoptosis

After indicated treatment, 200 µl Annexin V-FITC (C1062S-1, Beyotime Biotechnology) and 5 µl PI (C1062S-3, Beyotime Biotechnology) were handled with HK-2 cells for 15 min. Finally, the apoptotic cells were gauged using FACS Calibre flow cytometry (Accuri C6 Plus, BD Biosciences, USA).

### Detection of reactive oxygen species (ROS)

After indicated treatment, DCFH-DA (S0033S, Beyotime Biotechnology) was applied to handle with HK-2 cells for 20 min. Next, serum free medium was used to wash cells for three times, and then cells were analyzed by way of flow cytometry at 480 nm excitation wavelength and 525 nm emission wavelength.

### Statistical analysis

Data analyses were conducted by means of GraphPad Prism software (USA). *P* < 0.05 was supposed to statistical significant. All data were statistically compared using t-test or one-way analysis of variance (ANOVA).

## Supplementary Information

**Additional file 1.**

## Data Availability

The datasets used and analyzed during the current study are available from the corresponding author.
