# Supplementary material for: Knockdown of ELF4 aggravates renal injury in ischemia/reperfusion mice through promotion of pyroptosis, inflammation, oxidative stress, and endoplasmic reticulum stress
Source: BMC Mol Cell Biol. 2023 Jul 20;24:22. doi: 10.1186/s12860-023-00485-2 (PMC10360327; doi:10.1186/s12860-023-00485-2)
Supplement: Supplementary file 1 — Additional file 1. [file 12860_2023_485_MOESM1_ESM.docx]

Supplementary table 1

|  | Sequence of primers |
| --- | --- |
| ELF4 (mouse) | Forward: 5’- CAAAGATGGCAAAGGCAGCA -3’ |
|  | Reverse: 5’- CCTTCCACTTTGGCCAGGAT -3’ |
| GAPDH (mouse) | Forward: 5’- AGGTCGGTGTGAACGGATTT -3’ |
|  | Reverse: 5’- ACTGTGCCGTTGAATTTGCC -3’ |
| ELF4 (human) | Forward: 5’- CGCCGTCTCACCTGGTATTT -3’ |
|  | Reverse: 5’- CACAGGAGCGACCTCGG -3’ |
| GAPDH (human) | Forward: 5’- GCCGCATCTTCTTTTGCGTC -3’ |
|  | Reverse: 5’- CTTCCCGTTCTCAGCCATGTAG -3’ |
